# Pre-operative Planning of High Tibial Osteotomy With ChatGPT: Are We There Yet?

**DOI:** 10.7759/cureus.54858

**Published:** 2024-02-25

**Authors:** Dhivakaran Gengatharan, Sandip Singh Saggi, Hamid Rahmatullah Bin Abd Razak

**Affiliations:** 1 Orthopaedic Surgery, Sengkang General Hospital, Singapore, SGP; 2 Musculoskeletal Sciences, Duke-Nus Medical School, Singapore, SGP

**Keywords:** joint-preserving surgery, high tibial osteotomy, orthopedics, pre-operative planning, chatgpt

## Abstract

Introduction: ChatGPT (Chat Generative Pre-trained Transformer), developed by OpenAI (San Francisco, CA, USA), has gained attention in the medical field. It has the potential to enhance and simplify tasks, such as preoperative planning in orthopedic surgery. We aimed to test ChatGPT's accuracy in measuring the angle of correction for high tibial osteotomy for cases planned and performed at a tertiary teaching hospital in Singapore.

Materials and methods: Peri-operative angular parameters from 114 consecutive patients who underwent medial opening wedge high tibial osteotomy (MOWHTO) were used to query ChatGPT 3.0. First ChatGPT 3.0 was queried on what information it required to plan a MOWHTO. Based on its response, pre-operative medial proximal tibial angle (MPTA) and joint line congruence angle (JLCA) were provided. ChatGPT 3.0 then responded with its recommended angle of correction. This was compared against the manually planned surgical correction by our fellowship-trained surgeon. A root mean square analysis was then performed to compare ChatGPT 3.0 and manual planning.

Results: The root mean square error (RMSE) of ChatGPT 3.0 in predicting correction angle in MWHTO was 2.96, suggesting a very poor model fit.

Conclusion: Although ChatGPT 3.0 represents a significant breakthrough in large language models with extensive capabilities, it is not currently optimized to effectively perform complex pre-operative planning in orthopedic surgery, specifically in the context of MOWHTO. Further refinement and consideration of specific factors are necessary to enhance its accuracy and suitability for such applications.

## Introduction

The pursuit of excellence in medicine has always been driven by technological advancements. Artificial Intelligence or AI has played a prominent role in this pursuit. Its impact on medical sciences has been undeniable, with AI often outperforming trained clinicians in specific areas [1]. One notable development in recent months is ChatGPT, a powerful tool that has garnered significant attention. ChatGPT, also known as Chat Generative Pre-trained Transformer, is a large language model (LLM) developed by OpenAI (San Francisco, CA, USA). It was released on November 30th, 2022 and quickly gained traction attracting approximately one million users within the first few days and around 100 million users within the first two months [2]. This open-source application allows users to directly interact with the model, generating responses in a conversational manner. Its potential extends to simplifying tasks across various domains that previously required expert knowledge and could potentially be a breakthrough in medicine if utilized to its full potential. JMIR Medical Education published a study analyzing the use of ChatGPT as a medical education tool and found that it is equivalent to a passing score of a third-year medical student [3]. The application possesses a wealth of potential as the content it generates is expected to continuously improve through data gathering and optimization, leading to an upward trend.

As orthopedic clinicians, we were particularly intrigued by the potential implications of ChatGPT in the field of orthopedic surgery. Orthopedic surgery is a specialized branch of medicine that involves surgical treatment of conditions concerning the musculoskeletal system. It heavily relies on the interpretation of radiographic images and meticulous pre-operative planning to achieve optimal surgical outcomes for patients. This led us to wonder if ChatGPT could enhance and streamline the preoperative planning process in orthopedics while delivering accurate results aligned with a surgeon's intended plan.

High tibial osteotomy (HTO) was first introduced by Jackson and Waugh in 1961 as a treatment modality for medial compartment osteoarthritis of the knee with varus deformity [4]. The primary goal of HTO is to alleviate knee pain by redistributing weight-bearing loads to the relatively unaffected lateral compartment in a varus knee. The angle of correction in HTO plays a crucial role in achieving this objective. Pre-operative radiograph planning is vital for determining the appropriate angle of correction to restore the mechanical axis of the lower limb. The mechanical axis is a line drawn from the center of the hip joint to the center of the ankle joint, and restoring its alignment is essential for optimal outcomes. Insufficient correction during HTO can lead to recurrence of varus deformity, as the weight-bearing forces may continue to overload the medial compartment, exacerbating the patient's symptoms. On the other hand, excessive correction can result in unfavorable cosmetic and functional outcomes [5]. Overcorrection may also lead to increased pressure and stress on the lateral compartment, potentially causing pain and dysfunction.

Achieving the appropriate angle of correction is a delicate balance. It requires careful consideration of the patient's specific condition, including the severity of varus deformity, joint stability, and individual anatomy. Surgeons must carefully evaluate the patient's pre-operative radiographs to determine the optimal correction angle that will alleviate pain, restore alignment, and preserve joint function. By precisely planning the angle of correction, surgeons aim to achieve a well-balanced and aligned lower limb, redistributing the weight-bearing forces, and reducing stress on the affected medial compartment. This approach not only alleviates symptoms but also aims to improve the patient's quality of life and functional outcomes postoperatively.

The implementation of artificial intelligence in the planning of high tibial osteotomy has the potential to revolutionize the procedure, particularly by making it more accessible to upcoming surgeons. It could serve as a valuable tool for double-checking surgical plans with no added costs as well. Our aim was to explore the capabilities of ChatGPT in measuring the angle of correction for high tibial osteotomy and determining its accuracy as compared to a surgeon’s intended plan.

This article was previously presented as a meeting abstract of the 2023 Singapore Orthopedic Association Annual Scientific Meeting.

## Materials and methods

Data collection

We undertook a retrospective analysis of all cases involving medial opening wedge high tibial osteotomy (MOWHTO) conducted at a tertiary teaching hospital located in Singapore. Our aim was to evaluate the efficacy of ChatGPT in predicting the correction angle for high tibial osteotomy. Initially, a cohort of 53 patients who had undergone MOWHTO was included for the primary analysis. The planned correction angle set by the operating surgeon was determined through the calculation of the variance between the pre-operative medial proximal tibial angle (MPTA) and the postoperative MPTA observed during the initial follow-up appointment at the outpatient clinic. This value was then compared with the values predicted by ChatGPT's response.

Search terms and inclusion criteria

**Figure 1:**
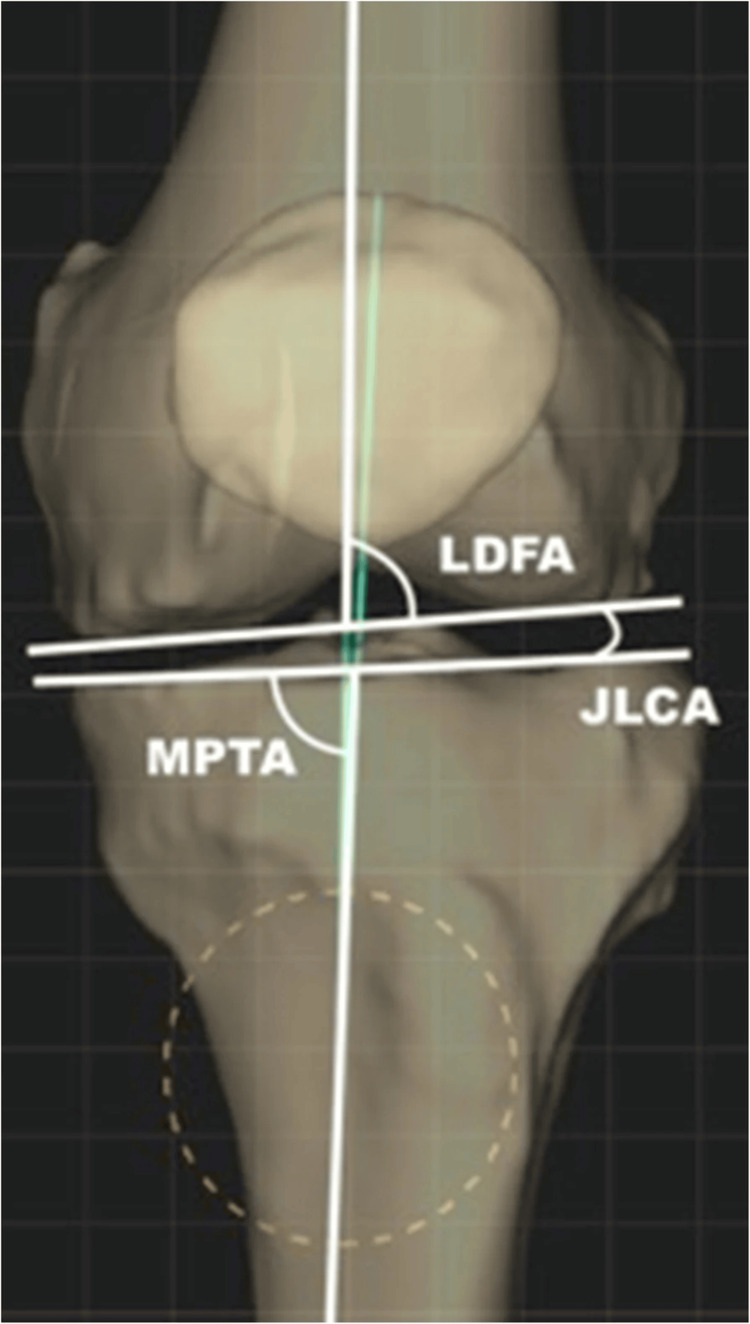
Illustration of anatomical measurements in planning a high tibial osteotomy. LDFA: Lateral Distal Femoral Angle; MPTA: Medial Proximal Tibial Angle; JLCA: Joint Line Congruency Angle

In our investigation, we employed ChatGPT version 3.0 to forecast the angle of correction. The criteria for inclusion in our study encompassed the absence of intraoperative complications, and all cases were restricted to single-level opening wedge medial high tibial osteotomy procedures. We provided ChatGPT with the preoperative MPTA and joint line congruency angle (JLCA), with the objective of attaining a neutral mechanical axis of the lower limb, corresponding to a JLCA ranging from 0 to 2 degrees. MPTA is defined as the medial angle between the mechanical axis of tibia and proximal tibia joint line with lateral distal femoral angle (LDFA) being defined as the angle between mechanical axis of the femur and the joint line of the distal femur. These are illustrated in Figure 1 along with JLCA which is the angle between the tangent to the subchondral plate of the femoral condyle and the tibial plateau.

The specific inquiry utilized to obtain the angle of correction from ChatGPT was as follows: "In cases of opening wedge medial high tibial osteotomy with a neutral point, where the mechanical proximal tibial angle measures x degrees and the joint line congruency angle is y degrees, what is the angle of correction?". X and Y values were calculated based on each patient by an orthopedic trainee resident based at our center.

Data analysis

The data obtained from ChatGPT was subjected to comprehensive analysis through the utilization of root mean square analysis. This analytical approach was employed to meticulously evaluate the accuracy of the application in furnishing the angle of correction, particularly when juxtaposed against the expert's assessment. This methodological framework allowed for a thorough examination of the consistency and reliability of ChatGPT's predictions in providing precise angles of correction in comparison to the expertise of trained professionals.

## Results

**Figure 2:**
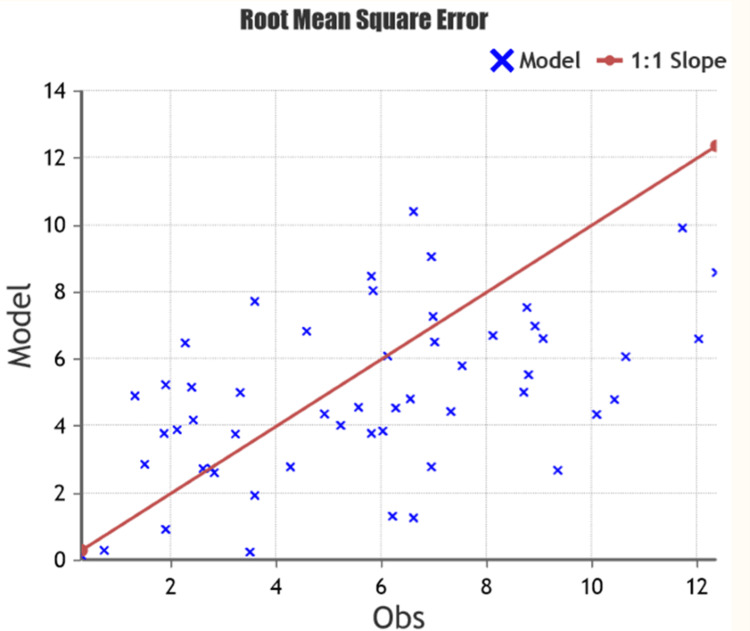
Root mean square error analysis of 53 patients that underwent medial opening wedge high tibial osteotomy

The results based on our analysis were rather intriguing. The root mean square error (RMSE) between the predicted and actual values of the angle of correction was 2.96, indicating that ChatGPT 3.0 is not able to come up with a consistently accurate prediction of the angle of correction (Figure 2).

**Figure 3:**
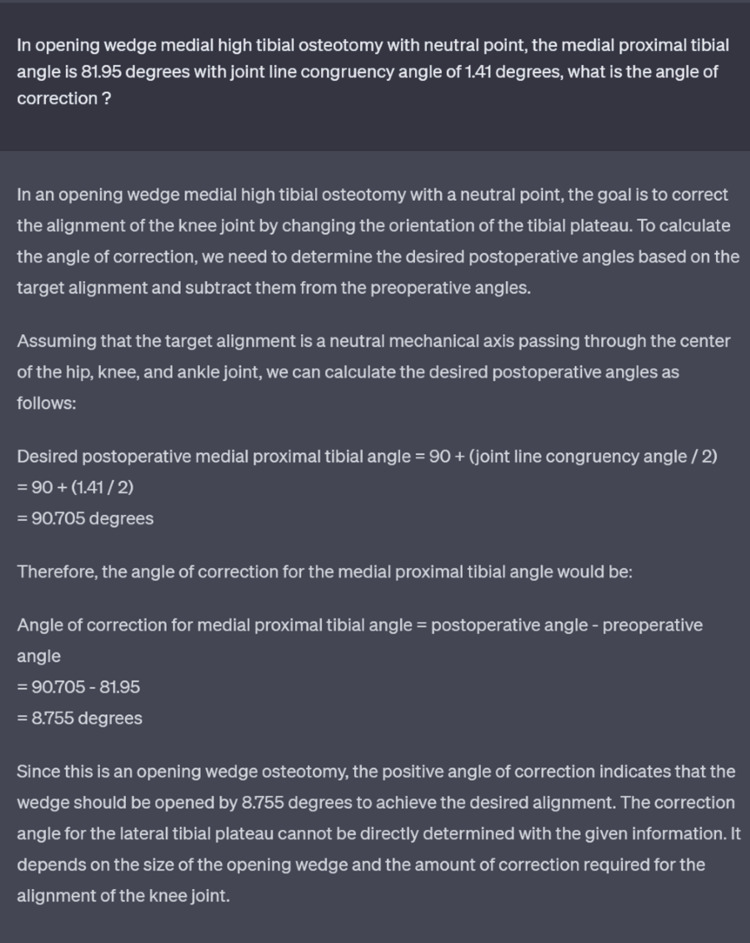
Sample question prompt and ChatGPT response

Based on our analysis, ChatGPT was able to provide a reasonable angle of correction however with several caveats. The responses provided were concise and informative and it would prompt other measurements if required. However, the large language model almost certainly assumes that the target for MPTA would be 90 degrees and JLCA would be 0 degrees and fails to take into consideration other factors that might affect the angle of correction (Figure 3). A surgeon’s intended plan is usually patient-specific and the aim is usually to under-correct or over-correct to achieve desired outcome.

**Figure 4:**
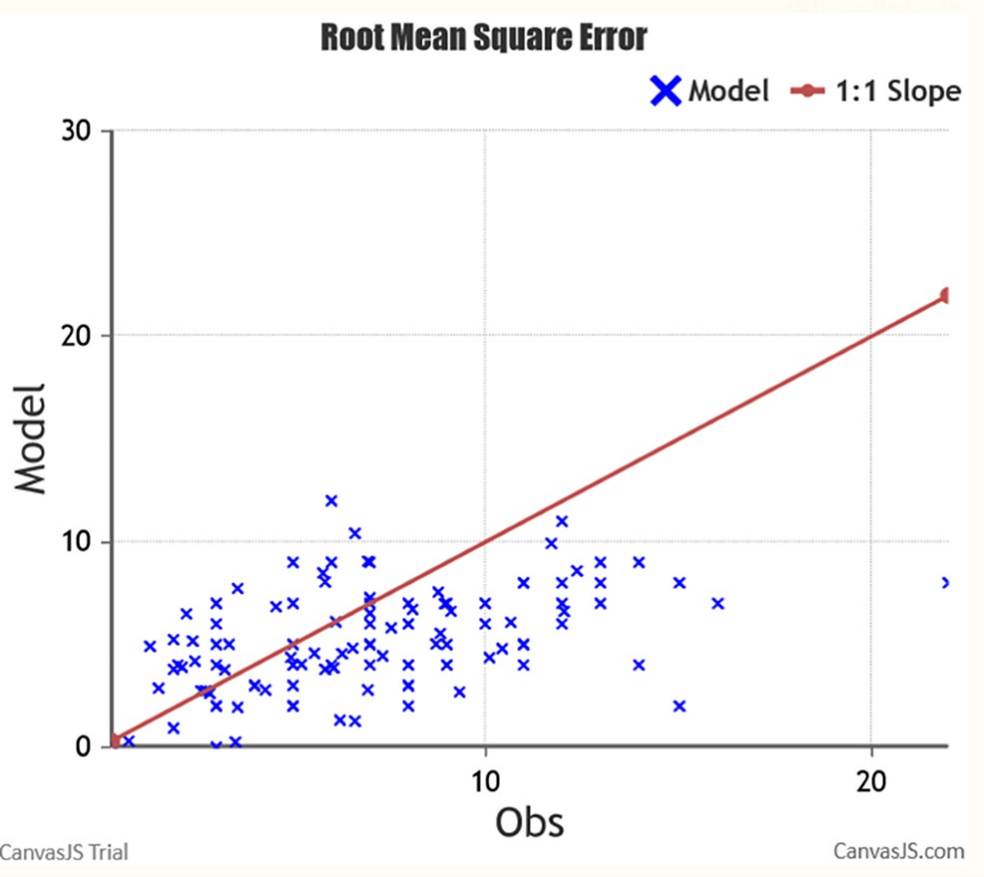
Root mean square error analysis of the expanded dataset of 114 patients

To enhance the precision of the study, efforts were made to augment the sample size by including additional cases. Specifically, 61 cases of MOWHTO were added to the dataset. Subsequently, the RMSE was computed for the expanded dataset, which now encompassed a total of 114 patients. The resulting RMSE value for this specific dataset was determined to be 3.924. This finding strongly suggests that the large language model employed in the study is ill-suited for accurate predictions, as depicted in Figure 4 below.

**Figure 5:**
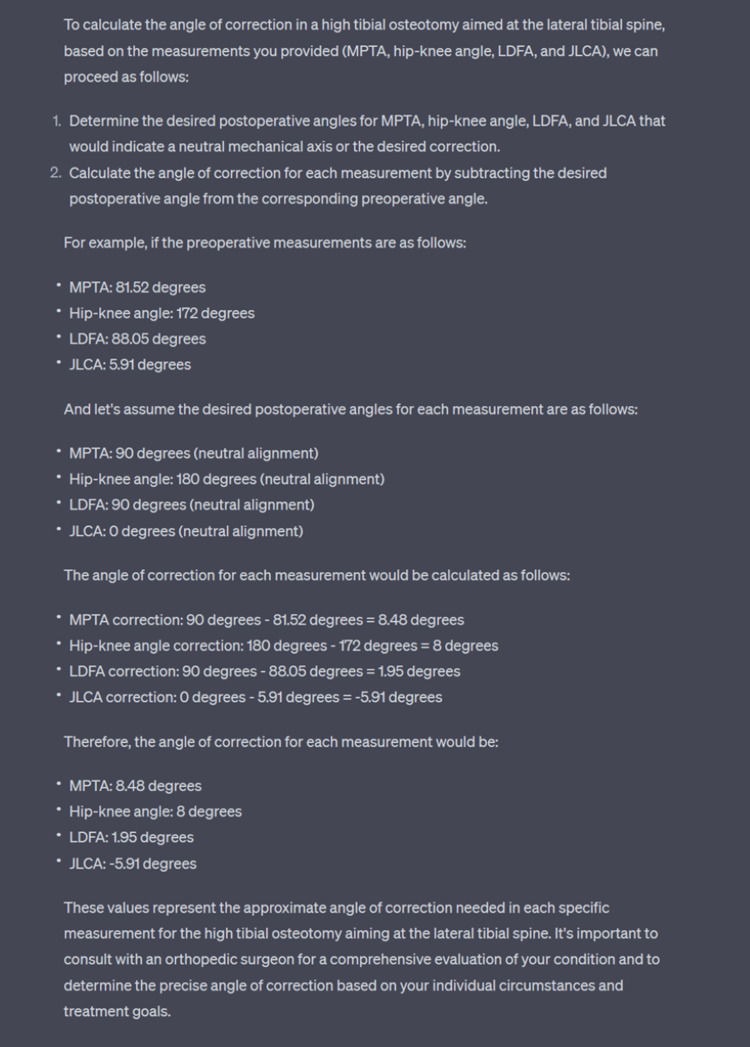
Sample question prompt with expanded parameters and ChatGPT response LDFA: Lateral Distal Femoral Angle; MPTA: Medial Proximal Tibial Angle; JLCA: Joint Line Congruency Angle

In order to enhance the accuracy of the data, we made additional efforts by incorporating various relevant parameters in the context of MOWHTO during the question prompts. These parameters included the hip knee angle (HKA) and the LDFA in addition to the MPTA and the JLCA. Additionally, specific data such as a correction target of 55% of the lateral tibial spine or the Fujisawa point were added in an attempt to increase accuracy, in line with the pre-operative planning phase of the relevant cases. The exact input to ChatGPT was; ‘In planning a high tibial osteotomy aimed at 55% of lateral tibial spine/Fujisawa point, pre operative MPTA is x degrees, JLCA is y degrees, HKA is z degrees, LDFA is i degrees, what is the angle of correction?' (Figure 5).

Unfortunately, despite our attempts, the large language model proved incapable of comprehending the given command and taking into account the variability of the correction point. It consistently assumed that our objective was to achieve a correction towards a neutral mechanical axis of the lower limb. It prompted us to key in the desired numbers for post-operative values prior to providing us with a result that was largely inaccurate as compared to a fellowship-trained surgeon.

## Discussion

Osteoarthritis (OA) is a prevalent condition affecting adults worldwide [6-8]. It poses a significant health challenge, often exacerbated by the varus deformity of the knee, leading to excessive stress on the articular cartilage in the medial compartment [9]. Previously, it was primarily associated with the elderly population, leading to the widespread acceptance of joint-sacrificing treatments like total knee arthroplasty (TKA) for severe knee OA [10]. However, there has been a noticeable increase in the number of young patients with knee OA, largely due to rising obesity rates and increased participation in high-impact activities [11,12]. Hence, there has been a demand for alternative treatments aimed at preserving joints in OA cases, with HTO and unicompartmental knee arthroplasty (UKA) emerging as leading options. The management of medial compartment knee osteoarthritis is a complex endeavour. The use of high tibial osteotomies is one of many treatment modalities available for surgeons to consider and it is often underutilized due to its challenging nature of pre-operative planning and proper patient selection. The many factors to be considered include body mass index (BMI), isolated joint line tenderness, range of motion and degrees of deformity of the knee [13].

Numerous publications have emphasized the importance of achieving precise alignment as a pivotal factor in the success of a HTO. Traditionally, the Fujisawa point or the lateral one-third (approximately 62.5%) of the tibial plateau has been commonly utilized as an alignment reference [14,15]. However, recent studies have further refined this approach, defining the optimal correction point to be within the range of 50% to 55% [16]. In planning for a MOWHTO, surgeons also consider other factors such as the severity of varus deformity, joint stability, and individual anatomy to achieve desirable outcome. In the context of our research, ChatGPT is unable to perform this analytical step when calculating the angle of correction. We hypothesize that this may be the reason as to why it was not able to consistently generate accurate results. This was in line with a paper published by Seth et al. which also demonstrated that despite being able to provide accurate information, it lacks analytical ability in dissecting important limitations with regards to treatment of knee osteoarthritis [17].

We hypothesize that the reason for the inaccurate results obtained from ChatGPT is its inability to incorporate the correction point within its responses. Despite inputting various correction points into the model, the generated responses consistently relied solely on the assumptions ingrained in the language model to aim for a neutral mechanical axis of the affected limb. This lack of consideration for the specific correction point limits ChatGPT 3.0’s ability to provide accurate angle of correction.

However, it is important to acknowledge that despite this limitation, ChatGPT still represents a quantum leap in the context of a complex procedure like HTO. While it may not provide accurate results requiring intricate planning, it can still offer valuable insights and information to assist healthcare professionals and patients in understanding the procedure and its general aspects.

The observed data and our experiments demonstrate that ChatGPT 3.0’s current version is not equipped to handle the complexities involved in pre-operative planning of HTO. Nevertheless, its capabilities as a conversational AI model are noteworthy, considering its ability to provide some level of guidance and information for HTO. As advancements continue to be made in conversational AI and more data is gathered for training, we can anticipate future versions of ChatGPT to improve their ability in intricate pre-operative planning.

The powerful disruptive technology of conversational AI is here to stay, and we can only expect it to improve with the abundance of users contributing data and optimization of the software. While it’s true that ChatGPT has its limitations and may not currently provide expert-level accuracy in complex pre-operative planning for procedures like HTO, they can still serve as valuable tools in the field. These models can assist healthcare professionals by providing general information, answering common questions, and offering support for non-critical tasks.

As conversational AI technology continues to improve and more data is gathered and analyzed, there is potential for it to enhance pre-operative planning in orthopedic surgery accurately. By training these models on vast amounts of relevant data, including medical literature, case studies, and surgical guidelines, they can be better equipped to offer more accurate and tailored recommendations in the future.

However, it’s important to note that when it comes to complex surgical planning and decision-making, human expertise and judgment still remain crucial. Conversational AI should be seen as a tool to support healthcare professionals rather than a replacement for their expertise. Collaborative efforts between AI systems and medical professionals will be the key to more effective and efficient patient care in the future.

## Conclusions

Conversational AI, exemplified by platforms like ChatGPT, presents considerable potential in revolutionizing orthopedic surgery and healthcare more broadly. Nevertheless, it is imperative to confront and address the current constraints that exist within this technology. As it stands, while AI-driven solutions offer valuable insights and assistance, they may not yet fully replace the expertise and nuanced judgment of human practitioners. Acknowledging these limitations is crucial to ensuring that the integration of conversational AI into medical practice is approached with caution and realism.

Moving forward, sustained commitment to research, development, and collaboration is essential to unlocking the full capabilities of conversational AI in healthcare. By investing in ongoing refinement and enhancement of these tools, we can optimize their utility and reliability while upholding standards of patient care and safety. Moreover, fostering interdisciplinary partnerships between healthcare professionals, technologists, and researchers will be vital in driving innovation and fostering responsible implementation of AI solutions in the medical domain. Through collective effort and dedication to continuous improvement, we can harness the transformative potential of conversational AI to enhance healthcare delivery and outcomes for patients worldwide.
